# Disposable Non-Enzymatic Glucose Sensors Using Screen-Printed Nickel/Carbon Composites on Indium Tin Oxide Electrodes

**DOI:** 10.3390/s151229846

**Published:** 2015-12-10

**Authors:** Won-Yong Jeon, Young-Bong Choi, Hyug-Han Kim

**Affiliations:** 1 Department of Nanobiomedical Sciences and BK21 PLUS NBM Global Research Center for Regenerative Medicine, Dankook University, Anseo-Dong, Cheonan, Chungnam 330-714, Korea; powerwy@hanmail.net; 2 Department of Chemistry, College of Natural Science, Dankook University, Anseo-Dong, Cheonan, Chungnam 330-714, Korea; chem0404@dankook.ac.kr

**Keywords:** nickel/carbon composite, biosensor, non-enzymatic glucose sensor, disposable glucose sensor

## Abstract

Disposable screen-printed nickel/carbon composites on indium tin oxide (ITO) electrodes (DSPNCE) were developed for the detection of glucose without enzymes. The DSPNCE were prepared by screen-printing the ITO substrate with a 50 wt% nickel/carbon composite, followed by curing at 400 °C for 30 min. The redox couple of Ni(OH)_2_/NiOOH was deposited on the surface of the electrodes via cyclic voltammetry (CV), scanning from 0–1.5 V for 30 cycles in 0.1 M NaOH solution. The DSPNCE were characterized by field-emission scanning electron microscopy (FE-SEM), X-ray photoelectron spectroscopy (XPS), and electrochemical methods. The resulting electrical currents, measured by CV and chronoamperometry at 0.65 V *vs.* Ag/AgCl, showed a good linear response with glucose concentrations from 1.0–10 mM. Also, the prepared electrodes showed no interference from common physiologic interferents such as uric acid (UA) or ascorbic acid (AA). Therefore, this approach allowed the development of a simple, disposable glucose biosensor.

## 1. Introduction

Glucose is used as an energy source by most organisms, from bacteria to humans. In particular, glucose is a key source of human energy, providing ~3.75 kilocalories of food energy per gram [1]. However, high blood glucose levels can lead to diabetic complications such as diabetic retinopathy, kidney damage, heart disease, stroke, neuropathy, and birth defects [2,3]. Furthermore, low concentrations of glucose in the blood can cause seizures, accidents, possible brain damage, and even death [4,5,6]. For these reasons, glucose sensing in humans is very important. To measure glucose levels, various detection methods such as HPLC [7], polarimetry [8], Raman spectroscopy [9], laser photoacoustic spectroscopy [10], near infrared spectroscopy [11], optical coherence tomography [12], and electrochemistry [13] have been applied. Among these, electrochemical methods have been used for the determination of glucose owing to their good sensitivity, reproducibility, and easy maintenance, as well as low cost [14].

An electrochemical glucose biosensor was first reported by Clark and Lyons in 1962 [15]. Since then, the devices have evolved through three major generations. First-generation glucose sensors measured hydrogen peroxide, which reduces oxygen by action of the enzyme inner FAD(Flavin adenine dinucleotide). However, these sensors suffer from two major problems: they are sensitive to the presence of electro-active interfering species in the blood, and they depend on free oxygen as a catalytic mediator. Second-generation glucose sensors used electron transfer from the enzyme to the electrode through a mediator. Unfortunately, the mediator still has the problem of shuttling the electrons from the enzyme to the electrode and this can cause a buildup of hydrogen peroxide. In third-generation glucose sensors, the electrode is connected to the enzyme directly without any mediator. However, direct electron transfer between the redox center of the enzyme and the electrode can be hindered by the enzyme’s protein shell and its large size [16].

All three generations of enzyme-based glucose sensors also have enzyme-related drawbacks such as sensitivity to thermal and chemical conditions [17]. Therefore, non-enzymatic glucose sensors have been developed as alternatives, using materials such as Cu [18], Pt [19], Au [20], alloys [21], carbon nanotubes [22], and Ni-based electrodes [23] to detect glucose. Ni-based glucose sensors offer advantages such as low cost, suitability, and sensitivity [24]. In these sensors, glucose is directly oxidized by the redox couple of Ni(OH)_2_/NiOOH on the electrode surface in alkaline medium [25]. Ni-based non-enzymatic glucose sensors have been fabricated with the redox couple of Ni(OH)_2_/NiOOH on load-type or glassy carbon electrodes [24,25,26]. However, these electrodes are hard to fabricate, and non-disposable. Moreover, their preparations are usually multi-step (especially for cleaning) and time consuming.

In this study, to overcome the disadvantages of earlier non-enzymatic glucose sensors, we proposed a new disposable electrode in which nickel/carbon composites were screen-printed on an ITO substrate. The DSPNCE (“disposable screen-printed nickel/carbon composite on ITO electrodes”) were prepared by curing at 400 °C for 30 min. The Ni(OH)_2_/NiOOH redox couple was formed on the DSPNCE by electrochemical deposition in 0.1 M NaOH solution. The electrical currents of the DSPNCE were measured by cyclic voltammetry (CV) and chronoamperometry. The DSPNCE showed high sensitivity, excellent electrochemical response, and specificity in the presence of interferents such as UA and AA.

## 2. Experimental Section

### 2.1. Chemicals and Reagents

Nickel paste was kindly donated by Daejoo Co. (Siheung, Korea). Carbon ink (423SS) was purchased from Acheson Colloids Co. (Port Huron, MI, USA). d-(+)-Glucose, UA, and AA were purchased from Sigma-Aldrich Co. (Milwaukee, WI, USA). Sodium hydroxide, hydrogen peroxide, and sulfuric acid were used without further purification. Deionized Milli-Q water (DW) (Millipore, Japan) was used for the preparation of solutions.

### 2.2. Preparation of the DSPNCE

**Scheme 1:**
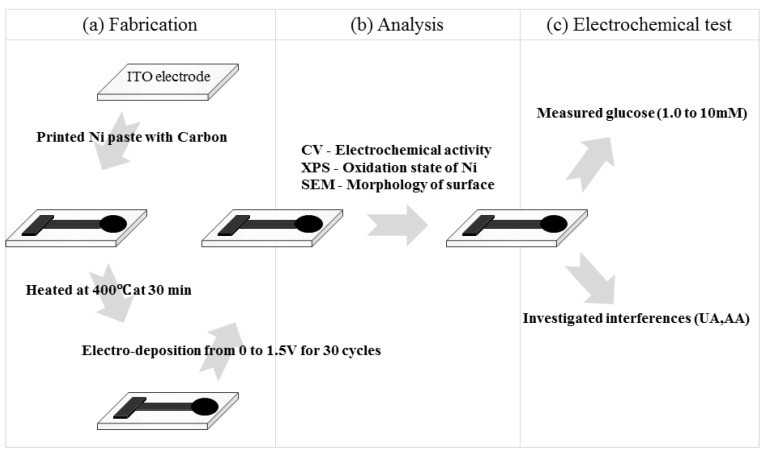
Schematic illustrating of the (**a**) fabricating of Ni(OH)_2_/NiOOH-DSPNCE; (**b**) analysis of Ni(OH)_2_/NiOOH-DSPNCE; and (**c**) the electrochemical test.

The ITO electrodes were cleaned by immersion in piranha solution (H_2_O_2_:H_2_SO_4_ = 3:1, *v*/*v*) for 5 min, rinsed and then sonicated for 5 min in DW, and finally, dried completely under a stream of nitrogen gas at room temperature. In a typical preparation, a composite of nickel paste and carbon ink (50:50 wt%) was screen-printed by a semiautomatic screen-printing machine (BS-860AP, Bando, Korea) on the ITO electrode. Other wt% composite ratios included 100:0, 75:25, 25:75, and 0:100. After drying for 12 h at room temperature, the electrode was heated in a muffle furnace (L3/11/P320, Nabertherm, Germany) at 400 °C for 30 min to remove the organic solvents and water. Other temperatures were also examined for this purpose (100, 200, 300, 400, 500, and 600 °C). Then, to form the Ni(OH)_2_/NiOOH on the DSPNCE, each electrode was subjected to cyclic voltammetry from 0–1.5 V *versus* Ag/AgCl for 30 cycles at a scan rate of 0.1 V/s in 0.1 M NaOH solution. Finally, the resulting electrode was used as the working electrode (Scheme 1a).

### 2.3. Spectroscopic Characterization of the DSPNCE Surface

The surface morphology of the DSPNCE was characterized using FE-SEM (Hitachi S-4300, Tokyo, Japan). Surface elemental quantification of the modified ITO electrode was performed by XPS (SPECS, Berlin, Germany) at 12 kV and 3 mA.

### 2.4. Electrochemical Measurements

An HP 34401A digital multimeter (Hewlett-Packard Company, Palo Alto, CA, USA) was used to check the resistance of the DSPNCE. All electrochemical measurements were performed using an electrochemical workstation (CHI660B, CH Instruments Inc., Austin, TX, USA) interfaced with a computer. A 6.0 mm diameter of DSPNCE was used as the working electrode. A micro Ag/AgCl (3.0 M KCl, Cypress, Lawrence, KS, USA) scrolled with a 0.5 mm diameter platinum wire was used as the reference and counter electrode, respectively. The electrical responses of the DSPNCE were measured by CV and chronoamperometry in 40 µL of glucose dissolved 0.1 M NaOH solution (Scheme 1b). CV and chronoamperometry experiments were performed to investigate the response to various glucose concentrations (0, 1.0, 2.0, 5.0, 7.5, and 10.0 mM) at 0.65 V *vs.* Ag/AgCl. To determine the possible interference by UA and AA, CV and chronoamperometry were investigated under the same conditions as for the glucose measurements. All the baseline CV and chronoamperometry results were corrected using CHI 660B software (CH Instruments, Inc., Bee Cave, TX, USA, Scheme 1c).

## 3. Results and Discussion

### 3.1. Characterization of the DSPNCE

#### 3.1.1. Resistance for the DSPNCE Prepared under Various Fabrication Conditions

Table 1 shows the measured resistance results for the DSPNCE prepared with various nickel/carbon ratios (100/0, 75/25, 50/50, 25/75, 0/100 *w*/*w*). For the DSPNCE screen-printed with 100 wt% nickel paste, the resistance could not be determined, and the DSPNCE prepared with 75 wt% nickel paste exhibited very high resistance (0.67 kΩ), which is unsatisfactory for use as an electrochemical biosensor. In the case of 50 wt% nickel paste, the resistance of the DSPNCE was 48 Ω, which is quite suitable for electrode use.

To remove organic solvents and water from the DSPNCE, the specimens were heated in a furnace at various temperatures (100, 200, 300, 400, 500, and 600 °C) (Table 2). The screen-printed nickel/carbon paste became detached from the ITO electrode at >500 °C, because the carbon paste is subject to ignition at this temperature [27]. Therefore, the optimum drying conditions for the DSPNCE were 400 °C for 30 min, which afforded a resistance value of 107 Ω. Although the resistance gradually decreased from 300–100 °C, the surface of the modified-ITO electrodes still retained organic solvent (Figure S1).

**Table 1:** Resistances of DSPNCE with Ni paste ratios.

Nickel (%)	Carbon (%)	Resistance (Ω)
100	0	N/A
75	25	670
50	50	48
25	75	34
0	100	22

**Table 2:** Resistance of curring temperatures.

Temperature (°C)	Resistance (Ω)	Note
Without Heating	48	
100	34	
200	42	
300	78	
400	107	
500	128	ITO was bent
600	21000	ITO was bent and carbon ink was detached

#### 3.1.2. Formation of Ni(OH)_2_/NiOOH on the DSPNCE

**Figure 1:**
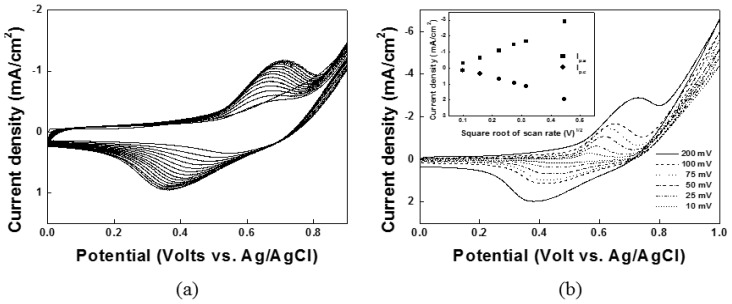
Cyclic Voltammograms for (**a**) electrodeposited Ni(OH)_2_/NiOOH on the DSPNCE and (**b**) at various scan rates from 10–200 mV/s; Inset of (**b**) was plotted by the anodic and cathodic peak currents *vs.* scan rate^1/2^.

To form the redox couple of Ni(II)/Ni(III), Ni(OH)_2_ and NiOOH were electrodeposited on the nickel/carbon composite on the DSPNCE using the CV technique, with 30 cycles from 0–1.5 V *vs.* Ag/AgCl in 0.1 M NaOH solution. In the first scan, the Ni(II)/Ni(III) redox peak on the modified ITO appeared at 0.538 V *vs.* Ag/AgCl (Figure 1a). The CV results show that Ni(II)/Ni(III) forms well on the nickel/carbon composite-modified ITO electrode. The electrochemical reactions of the Ni(II)/Ni(III) redox couple have been reported as follows [28]:

(1) $$\left[\mathrm{Ni}+{\mathrm{2OH}}^{-}\to \mathrm{Ni}{\left(\mathrm{OH}\right)}_{2}+\mathrm{2electrons}\;\mathrm{Ni}{\left(\mathrm{OH}\right)}_{2}+{\mathrm{OH}}^{-}\to \mathrm{NiOOH}+{\text{H}}_{2}\text{O}+\mathrm{electron}\right]$$

As shown in Figure 1b, the cyclic voltammogram of the electrodeposited Ni on the electrode in 0.1 M NaOH solution showed one pair of *quasi*-reversible redox peaks at *E*_1/2_ = 0.538 V *vs.* Ag/AgCl. The current density on the DSPNCE is shown in the inset of Figure 1b as a function of the square root of the scan rate; the anodic peaks ((*I*_p_)_a_) (r = 0.99462) and the cathodic peaks ((*I*_p_)_c_) (r = 0.99475) of the Ni(OH)_2_/NiOOH-DSPNCE increased linearly with the square root of the scan rate (*v*^1/2^) in the range of 0.01–0.2 V/s. These results suggest that the Ni(II)/Ni(III) redox couple on the DSPNCE undergoes a fast and reversible redox process, which is desirable for the investigation of glucose levels [29].

#### 3.1.3. Surface Characteristics of the DSPNCE

**Figure 2:**
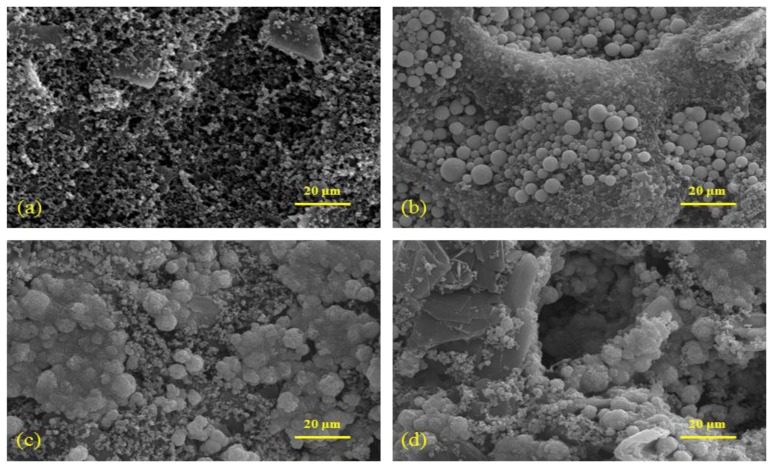
FE-SEM images (x10 K) of (**a**) pristine carbon ink printed ITO electrode, (**b**) printed 50 wt% nickel paste with carbon ink on the ITO electrode; (**c**) heated DSPNCE at 400 °C for 30 min and (**d**) electrodeposited Ni(OH)_2_/NiOOH on the DSPNCE.

The surface morphology of the DSPNCE was observed by FE-SEM. The typical FE-SEM images of the four types of samples, which were prepared with 50 wt % Ni paste on the DSPNCE, are shown in Figure 2. The image in Figure 2b clearly shows that the spherical nickel particles are widely covered with carbon ink on the modified-ITO electrodes. Figure 2c shows that the nickel particles are strongly adhered to the surface of the modified-ITO electrode after curing at 400 °C for 30 min. Figure 2d shows the development of a rougher morphology than that in Figure 2c, after CV was performed on the 400 °C-treated DSPNCE.

**Figure 3:**
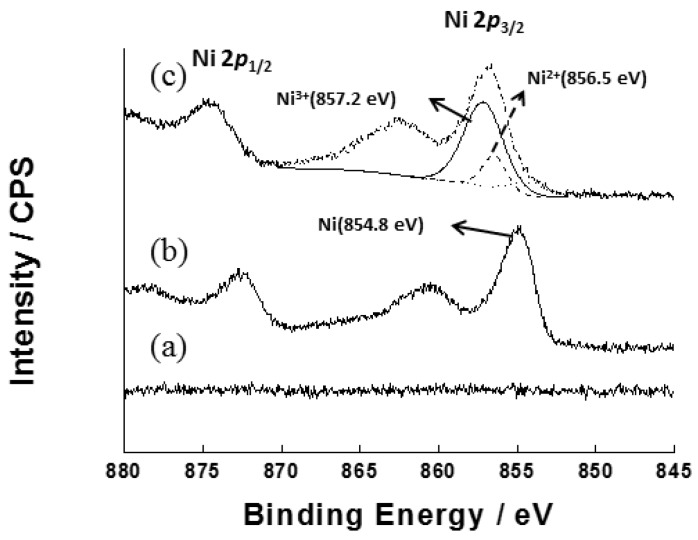
XPS spectra of Ni 2p of (**a**) bare ITO electrode; (**b**) heated DSPNCE at 400 °C for 30 min; and (**c**) electrodeposited Ni(OH)_2_/NiOOH on the DSPNCE after scanning 30 cycles in 0.1 M NaOH.

To verify the formation of Ni(OH)_2_ and NiOOH on the DSPNCE, XPS was performed. The wide-scan XPS analysis of the surface of the pristine ITO electrode shows no signals due to Ni (Figure 3a). Figure 3b reveals two distinct peaks for the zero-valent metallic state (Ni 2*p*_3/2_) at ~854.8 eV (2*p*_3/2_) and 872.7 eV (2*p*_1/2_), with a spin-orbit splitting of 17.9 eV. The spectrum obtained after the electrodeposition of Ni(OH)_2_/NiOOH on the DSPNCE shows two additional Ni 2*p*_3/2_ peaks at ~856.5 eV and 857.2 eV after 30 cycles of voltammetric scanning (Figure 3c), revealing the presence of Ni^2+^ and Ni^3+^. This XPS data agrees with previously reported data for Ni(OH)_2_ and NiOOH [28,30].

### 3.2. Electrochemical Measurements for Detecting Glucose

**Figure 4:**
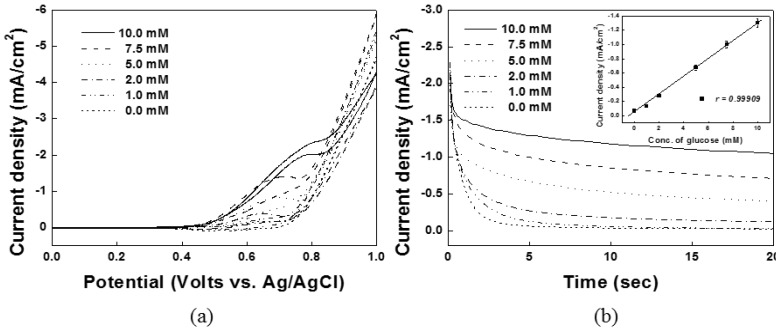
Electrochemical measurements of (**a**) cyclic voltamograms; and (**b**) chronoamperometric response with various glucose concentrations of 0, 1.0, 2.0, 5.0, 7.5 and 10.0 mM. Anodic currents at 0.65 V *vs.* Ag/AgCl was linearly increased with the concentration of glucose at inset of (**b**).

Cyclic voltammetric and chronoamperometric techniques were used to detect various concentrations of glucose. Figure 4a shows the CVs of the Ni(OH)_2_/NiOOH on the DSPNCE in the presence of various concentrations of glucose. The anodic peaks of Ni(OH)_2_/NiOOH increased linearly with successive increases in the glucose concentration (0, 1.0, 2.0, 5.0, 7.5, and 10.0 mM). Figure 4b shows that amperometric current responses were also obtained at various glucose concentrations. The data in the inset of Figure 4b was collected during chronoamperometry at 0.65 V *vs.* Ag/AgCl. The calibration plot of Figure 4b inset shows the linear dependence of the anodic peak current with the glucose concentration in the range of 1.0–10 mM, with a correlation coefficient of 0.99697. Also, the limit of detection (LOD) was determined as 0.4 mM, with a RSD of 4.75% (*N* = 10, where *N* denotes the number of different electrodes used). The catalytic current obtained from our new electrode was 1.3 mA/cm^2^ at 10 mM glucose, which is similar to previous reports for nickel-based electrodes in the direct measurement of glucose (Table 3).

**Table 3:** Comparisons of glucose performances of the Ni(OH)_2_/NiOOH-DSPNCE with the recently reported nickel-based non-enzymatic glucose sensor.

Electrode Matrix	Sensitivity (mA/cm^2^)	Linear Range (mM)	Applied Voltage (V)	Ref.
Ni(OH)_2_-CNT-PVDF	0.044399	0.25–39.26	0.55	[31]
Roselike a-Ni(OH)_2_	0.4188	0.00087–10.53	0.4	[32]
NiO-HMSs	2.39	0.00167–6.087	0.5	[33]
Ni(OH)_2_@oPPyNW	1.049	0.001–3.86	0.54	[34]
Ni(OH)_2_/ERGO-MWNT	2.042	0.01–1.5	0.54	[35]
Ni paste with carbon	1.3	1.0–10	0.65	This paper

### 3.3. Interference Studies of the DSPNCE

**Figure 5:**
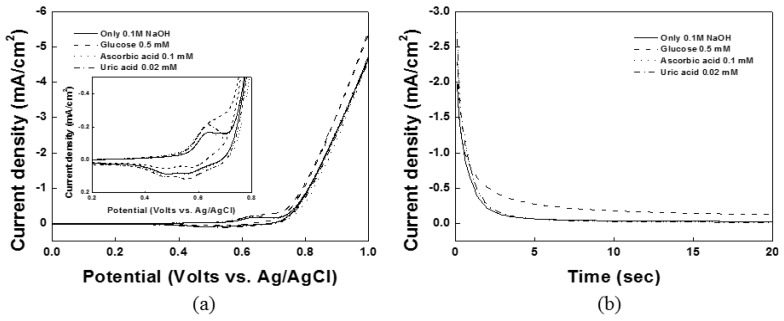
(**a**) Cyclic voltametric and (**b**) chronoamperometric response of glucose in the presence of 0.02 mM UA and 0.1 mM AA at DSPNCE in 0.1 M NaOH at 0.65 V *vs.* Ag/AgCl.

Interfering substances in physiological fluids (especially, UA and AA) can be oxidized along with the glucose in a non-enzymatic glucose sensor. As shown in Figure 5a,b, the effects of the interferents UA and AA on the Ni(OH)_2_/NiOOH-DSPNCE were studied in 0.1 M NaOH solution using CV and chronoamperometry. Based on the results in Figure 5b, no responses were observed for 0.02 mM UA or 0.1 mM AA compared to the baseline. Therefore, in the present work, the Ni(OH)_2_/NiOOH-DSPNCE shows good selectivity for the oxidation of glucose in the presence of physiological concentrations of UA and AA.

Also, the ability of our DSPNCE system to handle actual serum samples was shown by CV (Table 4). The glucose concentration for an actual sample measured with a commercial glucose sensor in a local hospital was 5.38 mM. Pure glucose was added to the serum sample at concentrations of 1 and 2 mM, and then measured by our sensor method (Figure S2).

**Table 4:** Glucose detection in serum by a commercial glucose sensor and our sensor method (*N* = 3).

Serum Sample	Commercial Glucose Sensor (mM)	Added Glucose (mM)	Our Sensor Method (mM)	RSD (%)
1	5.38			
		1.00	6.42	2.98
		2.00	7.48	3.51

## 4. Conclusions and Outlook

The important feature of the present work was the fabrication of a highly selective non-enzymatic glucose biosensor using disposable screen-printed nickel/carbon ITO electrodes (DSPNCE). The formation of Ni(OH)_2_/NiOOH on the electrode was confirmed by CV and XPS. The fully fabricated DSPNCE shows good electrochemical properties that are suitable for use as a non-enzymatic glucose sensor. Our DSPNCE exhibits several advantages, including easy fabrication, high sensitivity and selectivity, and excellent sensor performance. In addition, the oxidation of glucose by the Ni(OH)_2_/NiOOH on the electrode was not affected by the physiological interferents UA and AA. The screen-printable nickel paste for use in a non-enzymatic glucose sensor can be easily obtained, and its fabrication on various substrates such as glass, plastic, ceramic, and paper is currently under investigation in our laboratory.

## Supplementary Materials

The following are available online at http://www.mdpi.com/1424-8220/15/12/29846/s1, Figure S1: Thermo-gravimetric analysis (TGA) graph of the nickel/carbon composites at a 10 °C/min ramp rate. Figure S2: Cyclic voltammograms of added glucose in serum (line) and serum (dot) sample.
