# Supplementary material for: Disposable Non-Enzymatic Glucose Sensors Using Screen-Printed Nickel/Carbon Composites on Indium Tin Oxide Electrodes
Source: Sensors (Basel). 2015 Dec 10;15(12):31083–91. doi: 10.3390/s151229846 (PMC4721766; doi:10.3390/s151229846)
Supplement: Supplementary file 1 [file sensors-15-29846-s001.pdf]

Supplementary Materials: Disposable Non-Enzymatic Glucose Sensors Using Screen-Printed Nickel/Carbon Composites on Indium Tin Oxide Electrodes

Won-Yong Jeon, Young-Bong Choi and Hyug-Han Kim

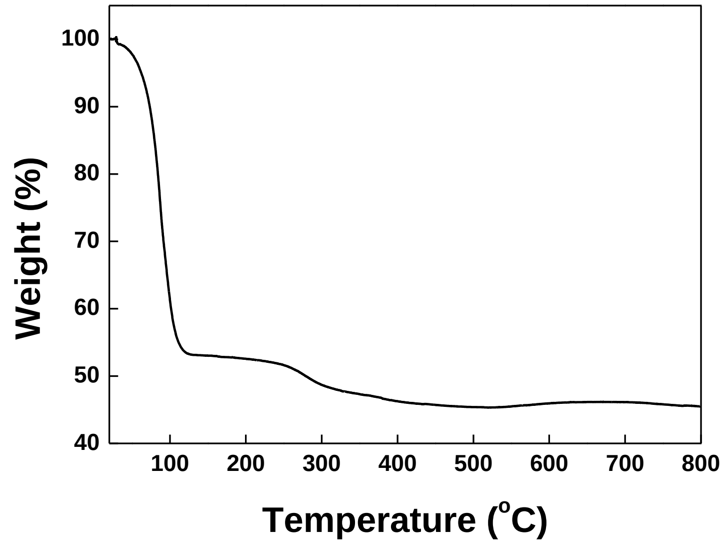

Figure S1: Thermo-gravimetric analysis (TGA) graph of the nickel/carbon composites at a 10 °C/min ramp rate.

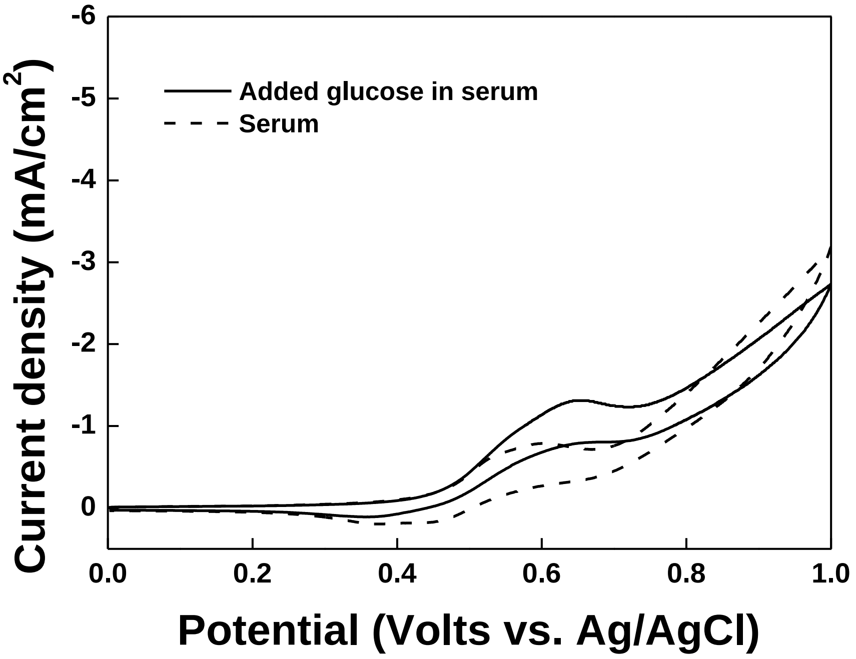

Figure S2: Cyclic voltammograms of added glucose in serum (line) and serum (dot) sample.
